# Changes in Human Meibum Lipid Composition Related to the Presence and Severity of Meibomian Gland Dysfunction

**DOI:** 10.1089/jop.2024.0063

**Published:** 2024-11-15

**Authors:** Ashley Nguyen, Kugen K. Naidoo, Layla Ajouz, Xiaoming Xu, Cathy Zhao, Michael R. Robinson, Douglas Borchman

**Affiliations:** ^1^ Allergan, an AbbVie company, Irvine, California, USA.; ^2^ Department of Ophthalmology and Visual Sciences, University of Louisville, Louisville, Kentucky, USA.

**Keywords:** disease severity, dry eye disease, lipid, meibum, meibomian gland dysfunction, nuclear magnetic resonance, biomolecular

## Abstract

**Purpose:**

Changes in meibum composition and quantity in meibomian gland dysfunction (MGD) result in tear film instability and dry eye. This exploratory study aimed to identify changes in (O-acyl)-ω-hydroxy fatty acid (OAHFA) and hydrocarbon chain (HC) unsaturation levels in meibum related to the presence and severity of MGD.

**Methods:**

Meibum samples were collected from 3 cohorts of adults with no MGD, mild-to-moderate MGD, and severe MGD in a noninterventional clinical trial (NCT01979887). OAHFAs, cholesterol esters (CE), HC unsaturation, and HC length in the meibum samples were quantified with ^1^H-nuclear magnetic resonance spectroscopy using 2 methods of normalization.

**Results:**

Meibum samples from 62 subjects were analyzed: 21 non-MGD, 21 mild-to-moderate MGD, and 20 severe MGD. Meibum OAHFA and CE levels and HC unsaturation were reduced with increasing severity of MGD, with most pairwise comparisons significant (*P* < 0.05, *t*-tests), following the order non-MGD > mild-to-moderate MGD > severe MGD. Regardless of the resonances used for normalization, each pairwise comparison of OAHFA, CE, and HC unsaturation levels in MGD (combined severities) versus non-MGD samples was significant (*P* < 0.01, *t*-test). Analysis using various normalization equations showed reductions of 20%–22% for OAHFAs, 51%–57% for CE, and 36%–66% for HC unsaturation in MGD (combined severities) compared with non-MGD. HC length was not altered in MGD (combined severities) compared with non-MGD samples (*t*-test).

**Conclusions:**

Meibum OAHFA, CE, and HC unsaturation levels were reduced in MGD and were lowest in the severe MGD cohort. These findings may contribute to the understanding of the pathophysiology of MGD.

## Introduction

Dry eye disease (DED) is a common multifactorial disease of the ocular surface characterized by loss of homeostasis of the tear film and accompanied by ocular symptoms including discomfort and visual disturbance.^1^ DED can be classified as aqueous-deficient, evaporative, or mixed DED.^1–7^ Current treatment options include topical lubricants, anti-inflammatory medications, heat therapy, intense-pulsed light therapy, and punctal occlusion.^8^

The tear film lipid layer (TFLL) constitutes the outer layer of the tear film on the ocular surface. The TFLL aids in the spreading of tears and ocular surface lubrication, serves as a barrier to evaporation, and stabilizes the tear film by reducing surface tension.^9–12^ The TFLL is largely formed by meibum secreted from the meibomian glands located in the eyelids.^13^ Meibum consists of a mixture of hundreds of lipids.^13,14^ Most of these lipids, such as the wax esters (WE) and cholesterol esters (CE) that represent the vast majority of the lipids in human meibum, are nonpolar.^13,14^ The polar lipids in meibum include (O-acyl)-ω-hydroxy fatty acids (OAHFAs) and free fatty acids.^14,15^ Within the TFLL, nonpolar lipids are believed to form the air–tear interface, while polar lipids lie beneath them and interface with the aqueous-mucin layer of the tear film.^16^ Alterations in the composition and organization of lipids in the TFLL are believed to contribute to tear film instability in meibomian gland dysfunction (MGD),^17–19^ which is a chronic disorder characterized by changes in the quantity and/or quality of secreted meibum^20^ and the most common cause of evaporative DED.^21^

The molar ratio of CE to WE (R_CE/WE_) affects the rheology of meibum lipids.^22^ Spectroscopic studies have demonstrated that R_CE/WE_ in meibum is decreased in individuals with MGD compared with control subjects, suggesting that changes in these meibum constituents may result from the pathophysiology associated with MGD and may impact the TFLL and tear film homeostasis.^19,23–25^ In our recent nuclear magnetic resonance (NMR) spectroscopic study of changes in meibum composition associated with MGD disease severity, decreases in R_CE/WE_ and increases in the molar ratio of aldehydes to WE (R_aldehyes/WE_) were largest in meibum samples from individuals with severe MGD.^19^

OAHFAs are amphiphilic lipids that constitute ∼3%–5% of meibum.^13,14^ A total of 196 unique species of OAHFAs have been detected in human meibum.^26^ OAHFAs have been proposed to stabilize the tear film by forming an interface between the outer nonpolar lipid layer of the TFLL and the aqueous-mucin layer of the tear film.^27^ They may also have a direct effect on inhibiting the rate of tear evaporation.^28,29^ Studies have shown that films containing 100% OAHFA inhibit the rate of evaporation *in vitro*^28^ and are as effective in inhibiting evaporation as a multilayer film of OAHFA and CE.^29^

In a study using mass spectrometry, several species of OAHFA were observed to be differentially expressed in the meibum and tears of individuals with MGD compared with normal control subjects, with all but one of the OAHFAs at lower levels in the individuals with MGD.^30^ Across all study participants, significant associations were shown between the meibum levels of several distinct OAHFAs and the rate of tear film thinning (evaporation); however, similar associations between OAHFAs in tears and the rate of tear film thinning were not evident.^30^ In another study, a significant decrease in OAHFA levels in meibum was measured in patients with type 2 diabetes mellitus and DED compared with normal control subjects.^31^ A study of the meibum composition in Asian patients with DED and control subjects also showed reduced OAHFA levels in the meibum of patients with DED compared with the control subjects.^32^ In the latter study, levels of several distinct species of OAHFA in meibum decreased with the severity of the DED, with levels of the OAHFAs in the meibum of patients with severe DED significantly lower than those in the meibum of patients with mild DED.^32^

It is well established that hydrocarbon chain (HC) unsaturation (more double bonds) is the major factor contributing to the phase transition temperature and order (fluidity) of lipids.^33^ A higher level of HC unsaturation is associated with greater disorder and fluidity of lipids. A phase transition occurs when lipids go from a disordered liquid crystalline phase to an ordered gel phase. The order and level of HC unsaturation of meibum are important because more ordered (stiffer, less unsaturated) lipids may clump, block the meibomian glands, and inhibit meibum spreading on the ocular surface. Catalytic saturation of human meibum and tear lipids has been shown to result in thicker and less elastic meibum and tear lipid films, with the effects being proportional to the level of unsaturation.^33–35^ Few studies have quantified HC unsaturation in meibum. Principal component analysis of meibum infrared spectra indicated that HC unsaturation is lower in meibum from donors with MGD,^36^ which could account for the increased lipid order^35^ and decreased flow of meibum in MGD. However, another study using Raman spectroscopy did not confirm this finding.^37^

There have been few studies of changes in the molecular composition of meibum related to MGD disease severity. The purpose of this exploratory study was to evaluate changes in OAHFA and HC unsaturation levels in meibum related to the presence and severity of MGD. This study used the same meibum samples used in our previous report on changes in meibum R_CE/WE_ and R_aldehydes/WE_ associated with MGD disease severity.^19^

## Methods

This analysis used meibum samples collected from individuals with no MGD, mild-to-moderate MGD, and severe MGD in a prospective, multicenter, noninterventional clinical study (ClinicalTrials.gov identifier: NCT01979887). The study design and criteria for assignment of participants into the non-MGD, mild-to-moderate MGD, and severe MGD cohorts were reported previously.^19,38,39^ Determination of the presence and severity of MGD was made on the basis of maximum meibum quality score for meibum quality among the 6 central meibomian glands in the lower lid, the sum of scores of the worst 2 symptoms on an ocular symptom questionnaire, and Schirmer test scores. The study was conducted in accordance with the principles of the Declaration of Helsinki, and the study protocol was approved by an institutional review board (IRB)/ethics committee (Quorum IRB or the UK National Health Service Health Research Authority) at each of the three study sites. All participants provided written informed consent.

### NMR analysis of meibum samples

Meibum was collected from study participants at the exit visit, prepared for NMR analysis, and analyzed with NMR as described previously.^19^ Briefly, a Meibomian Gland Evaluator (Johnson & Johnson Vision, Irvine, CA) was used to apply uniform pressure on the lower eyelid, and the meibum expressed from the 6 central meibomian glands was collected for the left and right eyes separately using Sebutape^®^ (Clinical and Derm, Dallas, TX) and meibum collection kits. Samples collected from the study eye were stored at −20°C. For NMR analysis, the Sebutape was placed into a 9-mm prelabeled microvial with a Teflon^®^ cap (Microliter Analytical Supplies, Suwanee, GA). Then the vial was filled with argon gas and 0.5 mL deuterated chloroform (Sigma-Aldrich, St. Louis, MO), sonicated for 10 min, and the solution was then transferred to a glass NMR tube (Sigma-Aldrich) for NMR analysis.

Spectra were acquired at 25°C with a minimum of 1,250 scans, 45° pulse width, and a relaxation delay of 1.000 s over ∼1.5 h using a Varian VNMRS 700 MHz NMR spectrometer (Varian, Lexington, MA) equipped with a 5 mm ^1^H[13C/^15^N] ^13^C enhanced pulsed field gradient cold probe (Palo Alto, CA). A solution containing 5 µL tetramethylsilane (Sigma-Aldrich) per mL deuterated chloroform was used as a standard, and tetramethylsilane was used as a 0 ppm reference. The CDCl_3_ resonance at 7.24 ppm was used to confirm the shift value of the samples and standard. Phasing, curve fitting, and integrating were performed with GRAMS 386 software (Galactic Industries Corp., Salem, NH).

### Measurement of CE

**FIG. 1.**
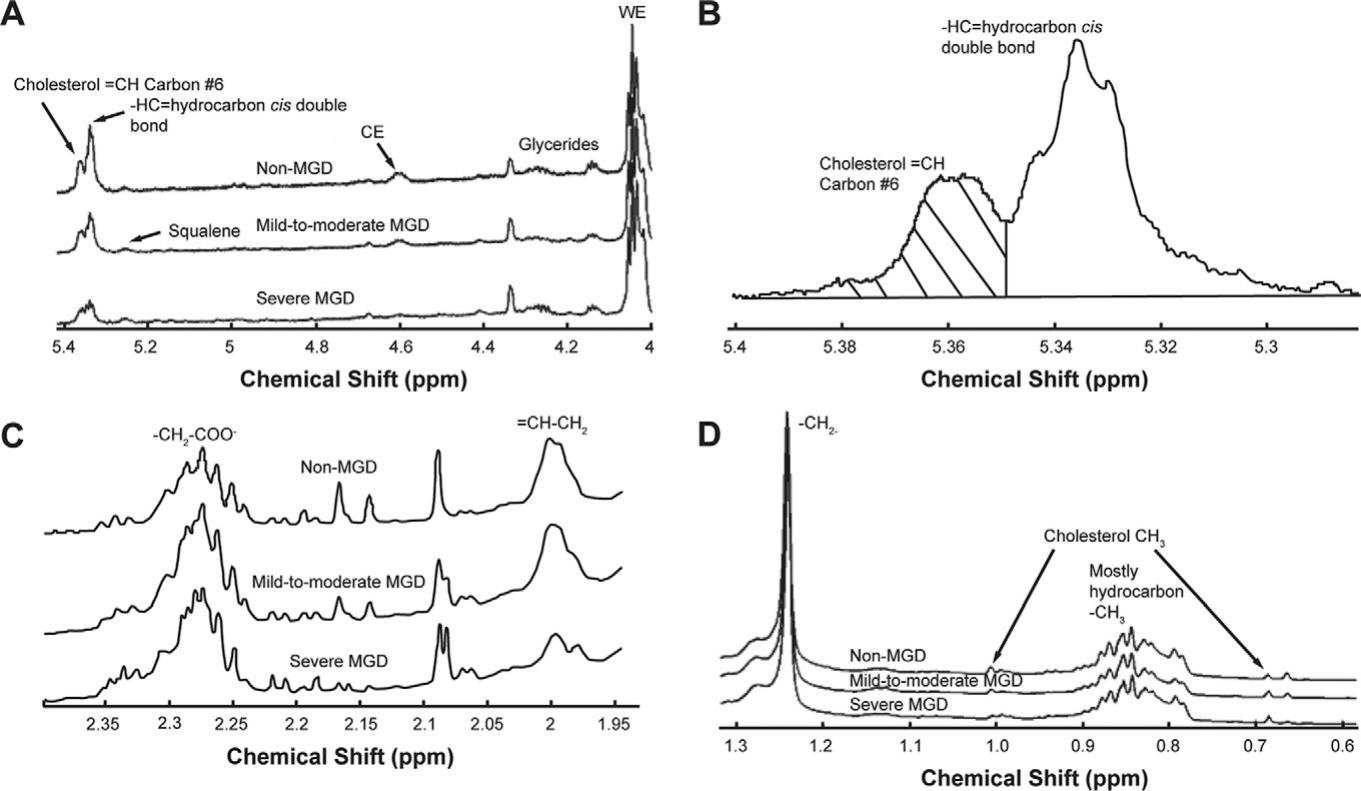
NMR spectra of meibum. The y-axis unit is resonance intensity, and the spectra were scaled and shifted along the *y*-axis individually. **(A)** Average spectra of meibum samples in the cohorts. CE were measured using the resonance at 5.36 ppm associated with the =CH proton on carbon #6 of cholesterol and related molecules. WE were measured using the resonance near 4.0 ppm. The resonance at 5.33 ppm is assigned to *cis*-HC double bonds and was used to measure unsaturation. **(B)** A typical spectrum of a meibum sample. HC unsaturation was measured by subtracting the intensity of the 5.36 ppm resonance assigned to *cis*-HC double bonds from the total intensity of the resonances near 5.36 and 5.33 ppm. **(C)** Average spectra of meibum samples in the cohorts. The hydrocarbon resonance assignment is 2.29 ppm for fatty acids (-CH2-COO-) and 2.0 ppm for =CH-CH2 moieties. The resonance at 2.0 ppm, as well as the resonance at 5.33 ppm, was used to measure unsaturation. **(D)** Average spectra of meibum samples in the cohorts. The hydrocarbon resonance assignment is 1.25 ppm for -CH2-moieties. CE, cholesterol esters; HC, hydrocarbon chain; NMR, nuclear magnetic resonance; WE, wax esters.

Both cholesterol and CE have a =CH proton on carbon #6 that resonates at 5.36 ppm (Fig. 1A, B) in the ^1^H-NMR spectra of human meibum.^19^ Since there is very little cholesterol in human meibum,^19^ this resonance was used to measure the CE content of the meibum samples. To calculate the molar ratio (R) of CE relative to the sum of CE and WE and relative to hydrocarbons, the resonance intensity at 5.36 ppm was normalized to the total of the WE resonance at 4.0 ppm (Fig. 1A) and the cholesterol and CE resonances at 1 and 0.63 ppm (Fig. 1D) and was also normalized to the HC resonances at 2.29 ppm (Fig. 1C), 2.0 ppm (Fig. 1C), and 1.25 ppm (Fig. 1D), with the following equations: R_CE/(WE + CE)_ = I_5.36_/[(I_1.0_ + I_0.63_)/6 + (I_4.0_)/2] and R_CE/hydrocarbons_ = I_5.36_/(I_2.29_ + I_1.25_ + I_2.0_). Resonances at 2.29, 2.0, and 1.25 ppm are assigned to protons associated with fatty acids (-CH_2_-COO^−^), =CH-CH_2_ moieties, and -CH_2_- moieties, respectively.^33^

These equations for the measurement of molar ratios of CE relative to hydrocarbons and the sum of CE and WE are complementary to the equations previously used for the measurement of R_CE/WE_ in the same meibum samples.^19^ The previous analysis used different resonances for CE than the 5.36 ppm resonance used in the current study and also normalized to only the 4.0 ppm resonance in WE.^19^

### Measurement of HC unsaturation

The resonance at 5.33 ppm assigned to *cis*-HC double bonds, -CH=, was used to measure HC unsaturation (Fig. 1B). The intensity of the =CH resonance at 5.36 ppm from the proton on carbon #6 of cholesterol was subtracted from the total intensity of the resonances near 5.36 and 5.33 ppm. The intensity of the resonance at 5.33 ppm was then normalized to the sum of the intensities of the WE and CE resonances and to the hydrocarbon resonances for the analysis of CE. The following equations were used: R_unsaturation/(WE + CE)_ = I_5.33_/[(I_1.0_ + I_0.63_)/6 + (I_4.0_)/2] and R_unsaturation/hydrocarbons_ = I_5.33_/(I_2.29_ + I_1.25_ + I_2.0_).

The resonance at 2.0 ppm (Fig. 1D) assigned to =CH-CH_2_ moieties was also used to measure HC unsaturation. The following equations were used: R_unsaturation/(WE + CE)_ = (I_2.0_/2)/[(I_1.0_ + I_0.63_)/6 + (I_4.0_)/2] and R_unsaturation/hydrocarbons_ = (I_2.0_/2)/[(I_2.29_ + I_1.25_ + I_2.0_)/2].

### Measurement of OAHFAs

The resonances near 2.29 ppm are assigned to OAHFAs (Fig. 1C). The following equations were used for the evaluation of OAHFA levels in the meibum samples: R_OAHFAs/(WE + CE)_ = I_2.29_/[(I_1.0_ + I_0.63_)/6 + (I_4.0_)/2] and R_OAHFAs/hydrocarbons_ = I_2.29_/(I_2.29_ + I_1.25_ + I_2.0_).

### Measurement of HC length

As resonances at 2.29, 2.0, and 1.25 ppm are assigned to HC protons in -CH_2_-COO^−^, =CH-CH_2_, and -CH_2_- moieties, respectively,^33^ the sum of the intensity of these resonances reflects HC length. The following equation was used to measure HC length relative to the amount of CE and WE: R_chain length/(WE + CE)_ = (I_2.29_/2 + I_2.0_/2 + I_1.25_/2)/[(I_1.0_ + I_0.63_)/6 + (I_4.0_)/2].

### Statistical analysis

Data are presented as mean ± standard error of the mean. Because this was an exploratory analysis, 2-sample *t-*tests assuming unequal variances were used to compare mean values between groups and provide *P* values for reference, without adjustment for multiplicity. As the clinical study was exploratory, the sample size was determined empirically, with a planned sample size of 25 participants in each cohort (75 total).^38^

## Results

As reported previously, the clinical study included 75 participants who were classified into the non-MGD (*n* = 25), mild-to-moderate MGD (*n* = 25), and severe MGD (*n* = 25) cohorts.^19,38,39^ Most of the participants were females (50/75, 66.7%) and either Black (33/75, 44.0%) or White (23/75, 30.7%), and the mean age was 54.5 years.^19,38,39^

### NMR analysis of meibum composition

NMR spectra were obtained for the meibum samples from 73 study participants.^19^ The analyzable spectra for the present analysis represented meibum samples from 62 participants: 21 in the non-MGD cohort, 21 in the mild-to-moderate MGD cohort, and 20 in the severe MGD cohort.

### HC unsaturation

**FIG. 2.**
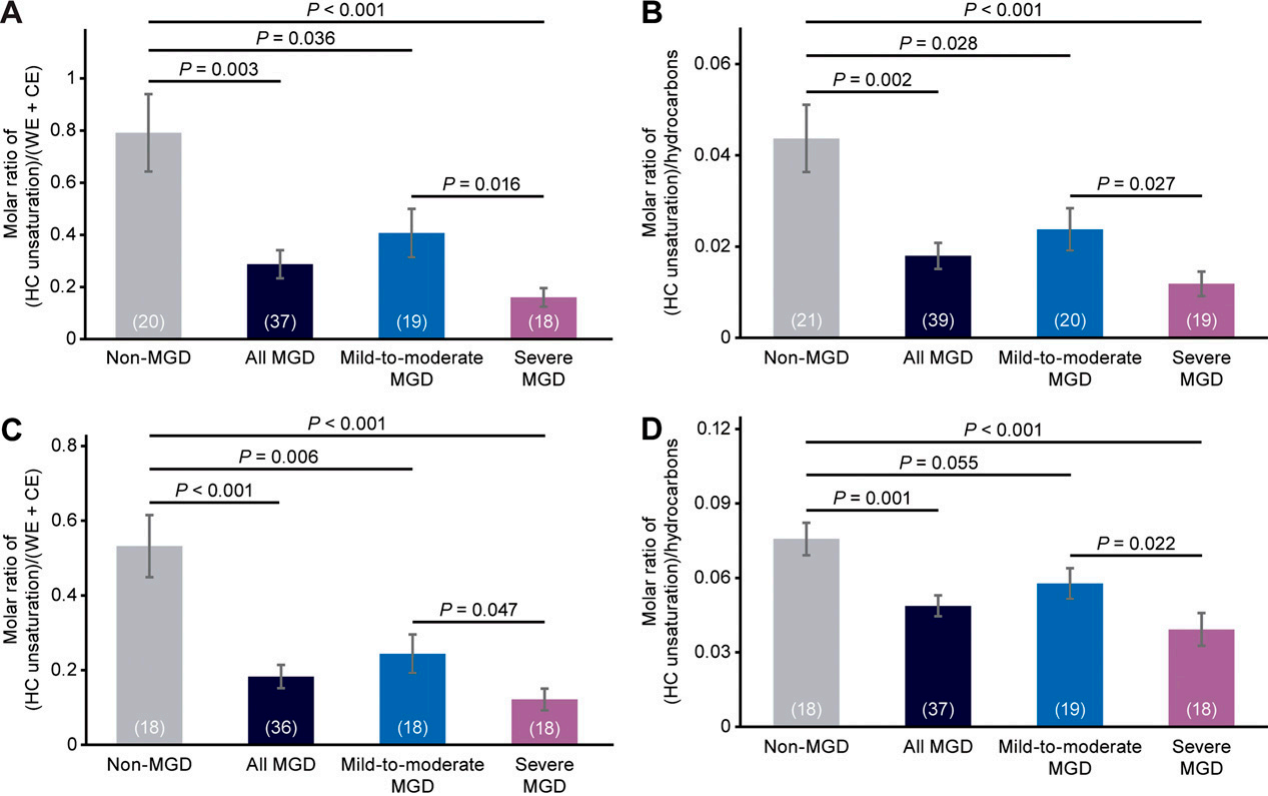
Analysis of meibum HC unsaturation levels in each cohort and in the combined mild-to-moderate and severe MGD cohorts (“all MGD”). Values in parentheses are the number of samples. Error bars show the standard error of the mean. The equations used to calculate the molar ratios were **(A)** R_(HC unsaturation)/(WE + CE)_ = (I_2.0_ / 2) / [(I_1.0_ + I_0.63_) / 6 + (I_4.0_) / 2]; **(B)** R_(HC unsaturation)/hydrocarbons_ = (I_2.0_ / 2) / [(I_2.29_ + I_1.25_ + I_2.0_) / 2]; **(C)** R_(HC unsaturation)/(WE + CE)_ = I_5.33_ / [(I_1.0_ + I_0.63_) / 6 + (I_4.0_) / 2]; and **(D)** R_(HC unsaturation)/hydrocarbons_ = I_5.33_ / (I_2.29_ + I_1.25_ + I_2.0_). I, resonance intensity at the indicated ppm; MGD, meibomian gland dysfunction; R, molar ratio.

The average NMR spectra differed among the 3 cohorts in the regions related to HC unsaturation (Fig. 1A, C). HC unsaturation levels consistently followed the trend non-MGD > mild-to-moderate MGD > severe MGD when analyzed with 4 different equations that used the intensity of different resonances for HC unsaturation (2.0 or 5.33 ppm) and normalized to the sum of the intensities of the WE and CE resonances or hydrocarbon resonances (Fig. 2). With each equation, HC unsaturation levels were significantly reduced in meibum samples from the combined mild-to-moderate and severe MGD cohorts compared with samples from the non-MGD cohort (Fig. 2), with the magnitude of the reduction ranging from 36% to 66% when HC unsaturation levels were analyzed with the 4 different equations.

Analysis with each equation showed lower HC unsaturation levels in the mild-to-moderate MGD samples than in the non-MGD meibum samples, as well as significantly lower HC unsaturation levels in the severe MGD cohort than in the mild-to-moderate MGD cohort (Fig. 2). The decrease in HC unsaturation levels in mild-to-moderate MGD compared with non-MGD samples ranged from 24% to 54%, and the decrease in HC unsaturation levels in severe MGD compared with mild-to-moderate MGD samples ranged from 32% to 61% when HC unsaturation levels were analyzed with the 4 different equations.

### CE content

**FIG. 3.**
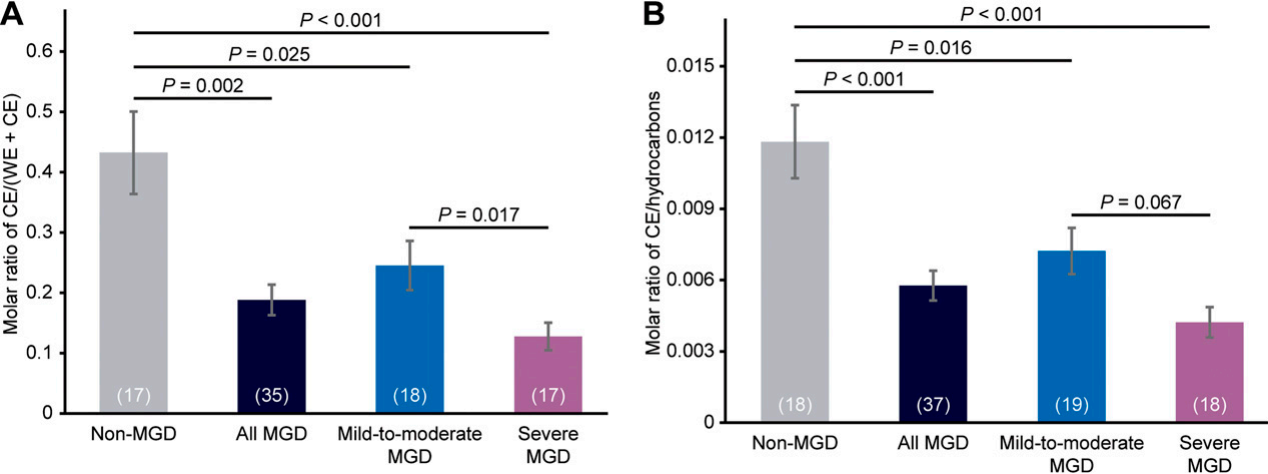
Analysis of meibum CE levels in each cohort and in the combined mild-to-moderate and severe MGD cohorts (“all MGD”). CE levels were normalized to **(A)** CE + WE and **(B)** hydrocarbon levels. Values in parenthesis are the number of samples. Error bars show the standard error of the mean.

As reported previously,^19^ there were significant differences in the average NMR spectra among the 3 cohorts in the regions related to the amount of CE (Fig. 1A). The 2 equations used in the current study to evaluate CE levels used a different resonance for CE than those used previously (the resonance at 5.36 ppm vs. those at 4.6 ppm, 1.0 ppm, and 0.63 ppm), as well as different resonances for normalization (normalizing to the sum of the intensities of the WE and CE resonances or to hydrocarbon resonances, rather than to the WE resonance). With each equation, meibum CE content followed the order non-MGD > mild-to-moderate MGD > severe MGD (Fig. 3). CE levels were significantly reduced in meibum samples from the combined mild-to-moderate and severe MGD cohorts compared with those from the non-MGD cohort by 57% (*P* = 0.002) when CE levels were normalized to the sum of WE and CE resonances and by 51% (*P* < 0.001) when CE levels were normalized to hydrocarbon resonances (Fig. 3). When normalized to the sum of WE and CE resonances, the CE content in the mild-to-moderate MGD samples was 43% lower (*P* = 0.025) than in the non-MGD samples, and the CE content in the severe MGD samples was 48% lower (*P* = 0.017) than in the mild-to-moderate MGD samples (Fig. 3). When normalized to hydrocarbon resonances, the CE content in the mild-to-moderate MGD samples was 39% lower (*P* = 0.016) than in the non-MGD samples, and the CE content in the severe MGD samples was 41% lower (*P* = 0.067) than in the mild-to-moderate MGD samples (Fig. 3).

### OHAFA content

**FIG. 4.**
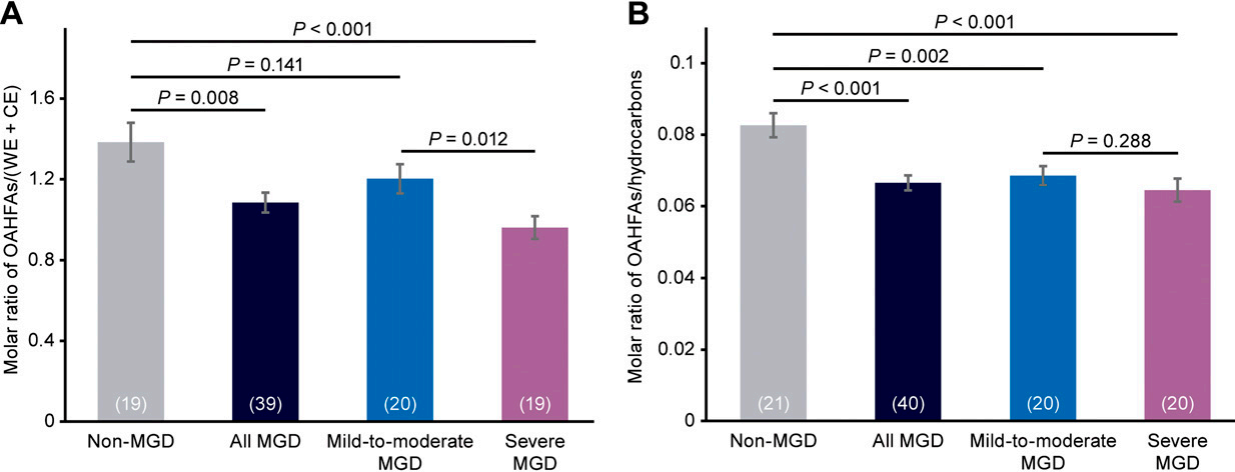
Analysis of meibum OAHFA levels in each cohort and in the combined mild-to-moderate and severe MGD cohorts (“all MGD”). OAHFA levels were normalized to **(A)** CE + WE and **(B)** hydrocarbon levels. Values in parentheses are the number of samples. Error bars show the standard error of the mean. OAHFA, O-acylated ω-hydroxy fatty acid.

Average NMR spectra showed differences among the 3 cohorts in the region related to OAHFA (Fig. 1c). Meibum OAHFA content followed the trend non-MGD > mild-to-moderate MGD > severe MGD (Fig. 4). The OAHFA content of meibum from individuals with MGD (the combined mild-to-moderate and severe MGD cohorts) was significantly reduced compared with the non-MGD cohort by 22% (*P* = 0.008) when OAHFA levels were normalized to the sum of WE and CE resonances and by 20% (*P* < 0.001) when OAHFA levels were normalized to hydrocarbon resonances (Fig. 4). When the OAHFA resonance was normalized to the sum of WE and CE resonances, OAHFA levels were 13% lower in the mild-to-moderate MGD cohort than in the non-MGD cohort (*P* = 0.141) and 20% lower in the severe MGD cohort than in the mild-to-moderate MGD cohort (*P* = 0.012) (Fig. 4). When the OAHFA resonance was normalized to hydrocarbon resonances, OAHFA levels were 17% lower in the mild-to-moderate MGD cohort than in the non-MGD cohort (*P* = 0.002) and 6% lower in the severe MGD cohort than in the mild-to-moderate MGD cohort (*P* = 0.288) (Fig. 4).

### HC length

**FIG. 5.**
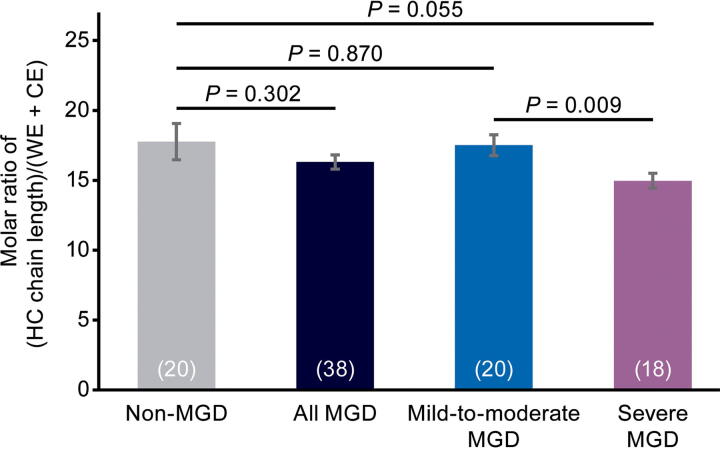
Analysis of meibum HC length in each cohort and in the combined mild-to-moderate and severe MGD cohorts (“all MGD”). HC length (number of carbon atoms) is normalized with respect to ester (CE + WE) content. Values in parentheses are the number of samples. Error bars show the standard error of the mean.

Meibum relative HC length normalized to the total CE and WE content was not significantly different between the non-MGD cohort and the combined mild-to-moderate and severe MGD cohorts, or between the non-MGD cohort and the mild-to-moderate MGD cohort (Fig. 5). However, relative HC length normalized to the total CE and WE content was significantly longer (*P* = 0.009) by 2.5 carbons in the mild-to-moderate MGD samples compared with the severe MGD samples (Fig. 5).

## Discussion

The composition of meibum affects the structure and function of the TFLL,^18^ and changes in meibum composition are characteristic of MGD^40^ and believed to contribute to the dry eye associated with the disease.^17–19^ Studies have shown alterations in meibum levels of polar lipids (such as OAHFAs^41^ and sphingolipids^42^) and nonpolar lipids (such as CE^23–25^) in MGD. However, the relationship between meibum compositional changes and MGD disease severity requires further clinical investigation. The current study showed that the presence and increased severity of MGD are associated with decreased OAHFA levels and decreased HC unsaturation in meibum. It also confirmed a relative reduction of CE levels in the meibum of individuals with MGD^23–25^ and our previous findings of progressively lower CE levels in meibum associated with increasing MGD disease severity.^19^

OAHFAs at the interface between the aqueous layer of the tear film and the TFLL may serve as a scaffolding, along with phospholipids, for the bulk WE and CE located superficially to them.^27^ A shortage of OAHFAs could potentially inhibit the spreading of the bulk WE and CE in the TFLL, leading to a patchy TFLL, tear film instability, and tear evaporation, and there is evidence suggesting that a decrease in OAHFAs (overall or in specific OAHFAs) may be involved in the etiology of DED.^30–32,41^ In a transgenic mouse model, deficiency in *Cyp4f39*, a gene encoding a fatty acid ω-hydroxylase, resulted in decreased levels of C16:1 OAHFAs, cholesteryl OAHFAs, and wax diesters in the meibomian glands, as well as obstruction of the orifices of the meibomian glands, a decrease in tear film breakup time, and corneal epithelial damage indicative of MGD and DED.^43^ Clinical study results have also suggested an association between reduced OAHFA levels and the presence^31^ or severity^32^ of DED. Moreover, a study of OAHFA levels in the tears of patients with MGD who underwent eyelid warming treatment showed that the treatment resulted in increases in total OAHFA levels and levels of several distinct OAHFA species, which correlated significantly with improvement in ocular discomfort.^44^

A previous, smaller study reported by Lam et al.^32^ showed a 16% decrease in OAHFA content (expressed as a percentage of total lipids) in the meibum of a mixed population of donors with evaporative and/or aqueous-deficient DED compared with donors without DED (controls: 3.458 ± 0.485, *n* = 10; patients with DED: 2.909 ± 0.266, *n* = 27).^32^ This decrease in meibum OAHFA content is similar to the 20%–22% decrease in meibum OAHFA levels measured in the current study in individuals with MGD compared with those without MGD. Consistent with our findings of progressively lower meibum OAHFA levels associated with increased severity of MGD, in the study by Lam et al.,^32^ total OAHFA levels were related to disease severity, following the trend mild DED > moderate DED > severe DED, with levels of several distinct species of OAHFA significantly higher in the patients with mild DED than in those with severe DED.^32^

An advantage of our ^1^H-NMR technique is that regardless of the HC lengths, saturation levels, and branching, all forms of OAHFA resonate at 2.29 ppm. Thus, the intensity of the 2.29 ppm resonance provides a measure of the total OAHFA level, including both major and minor species of OAHFA, without any need for purification or standards. On the contrary, ^1^H-NMR spectroscopy is unable to differentiate levels of individual species of OAHFA. A total of 196 distinct species of OAHFA have been detected in human meibum,^26^ and the meibum levels of distinct OAHFA species may be differentially regulated. A study using mass spectrometry showed that levels of several distinct OAHFAs were decreased in the meibum and/or tears of individuals with MGD compared with control subjects, but levels of one OAHFA (18:2/16:2) were increased.^41^ Moreover, decreased levels of several distinct OAHFAs were associated with an increased rate of tear film thinning/evaporation, but for one OAHFA (18:2/18:1), increased levels were associated with an increased rate of tear film thinning/evaporation.^30^ Thus, the effects of OAHFAs on tear evaporation and DED may be related to overall OAHFA levels in the meibum or tears, the mixture of OAHFAs present, or levels of distinct OAHFAs.

HC unsaturation is a key factor affecting the phase transition temperature and fluidity of lipids.^33^ Our previous study using infrared spectroscopy and principal component analysis showed a decrease in *cis* double bonds in the meibum of individuals with MGD compared with normal donors.^36^ The current study findings confirmed a decrease in HC unsaturation in meibum samples from individuals with MGD and further demonstrated a progressive decrease in meibum HC unsaturation associated with increasing disease severity. Using 4 different measures of HC unsaturation, we observed a 36%–66% reduction in double bonds in meibum from individuals with MGD compared with those without MGD. Based on the results of our catalytic saturation study,^34^ a 53% reduction in double bonds would cause human meibum to go from 68% disordered to 20% disordered and would increase the phase transition temperature from 30°C to 40°C. Thus, lipid unsaturation more than accounts for the difference in the lipid order of meibum between individuals with and without MGD.^45^

As a consequence of less unsaturation, meibum lipids become more ordered or stiff, which could inhibit the flow of meibum from the meibomian glands. It is reasonable to speculate that these more ordered lipids contribute to the formation of a discontinuous, patchy (more heterogenous) TFLL. This alteration in the structure of the TFLL is associated with deteriorated spreading and surface elasticity of the tear film, loss of TFLL function as an evaporative barrier, and the development of tear film instability and dry eye.^18^

As there was no significant difference between the MGD and non-MGD meibum samples in the relative HC length and no trend for increased HC length with increasing severity of MGD, changes in HC length are unlikely to contribute to increased viscosity of the meibum in individuals with MGD.

It is well established that R_CE/WE_ in meibum is decreased in MGD.^19,23–25^ As changes in R_CE/WE_ were shown to influence the rheology of tear lipids on an aqueous surface *in vitro*,^22^ it is likely that this change in meibum composition contributes to dry eye in MGD. Using the same meibum samples as in the current study, we demonstrated that R_CE/WE_ was reduced by ∼63% reduction in individuals with MGD compared with individuals without MGD, and the decrease in R_CE/WE_ was related to the severity of MGD.^19^ In the current study, CE levels were evaluated and normalized using different resonances than previously reported, and the results were consistent with the previous findings.^19^ Meibum CE levels were significantly reduced in the MGD samples compared with the non-MGD samples, and the largest decreases in CE content were observed in the meibum samples from individuals with severe MGD.

One limitation of this study is that levels of individual OAHFAs in the meibum samples could not be evaluated. Since the sample is not altered by ^1^H-NMR analysis, future studies could combine ^1^H-NMR spectroscopy with spectrometry techniques to evaluate changes in the levels of specific OAHFAs associated with MGD disease severity.

## Conclusions

This study used an NMR spectroscopic approach to evaluate meibum composition in participants with and without MGD. The results confirmed a significant decrease in CE content of meibum related to disease severity in individuals with MGD and showed that meibum OHAFA and lipid unsaturation levels are also altered in MGD and thus may contribute to the dry eye associated with the disease. The OHAFA content of meibum was significantly decreased in individuals with MGD, with OHAFA levels following the order non-MGD > mild-to-moderate MGD > severe MGD. A decrease in OHAFAs at the aqueous lipid layer interface in MGD could influence the spreading of the bulk layer above it, leading to a patchy, heterogeneous TFLL and tear instability. The results also showed a significant decrease in HC unsaturation related to disease severity in the meibum of individuals with MGD. The decrease in HC unsaturation is likely to influence HC order and the rheology of the TFLL, inhibiting the flow of meibum from the meibomian glands and potentially contributing to the formation of a discontinuous patchy TFLL and tear instability.

## Data Sharing Statement

AbbVie is committed to responsible data sharing regarding the clinical trials we sponsor. This includes access to anonymized, individual, and trial-level data (analysis data sets), as well as other information (e.g., protocols, clinical study reports, or analysis plans), as long as the trials are not part of an ongoing or planned regulatory submission. This includes requests for clinical trial data for unlicensed products and indications. These clinical trial data can be requested by any qualified researchers who engage in rigorous, independent, scientific research and will be provided following review and approval of a research proposal, Statistical Analysis Plan, and execution of a Data Sharing Agreement. Data requests can be submitted at any time after approval in the United States and Europe and after acceptance of this article for publication. The data will be accessible for 12 months, with possible extensions considered. For more information on the process or to submit a request, visit the following link: https://www.abbvieclinicaltrials.com/hcp/data-sharing/.
